# Integrating Genetic Data and Electronic Medical Records to Reassess Variant Pathogenicity in the Taiwanese Han Population

**DOI:** 10.3390/genes17070818

**Published:** 2026-07-17

**Authors:** Wei-De Lin, Ting-Yuan Liu, Yu-Chia Chen, Chi-Chou Liao, Fuu-Jen Tsai

**Affiliations:** 1 Department of Medical Research, China Medical University Hospital, Taichung 404327, Taiwan; 006203@tool.caaumed.org.tw (W.-D.L.);; 2 School of Post Baccalaureate Chinese Medicine, China Medical University, Taichung 404333, Taiwan; 3 Million-Person Precision Medicine Initiative, Department of Medical Research, China Medical University Hospital, Taichung 404327, Taiwan; 4 School of Chinese Medicine, China Medical University, Taichung 404333, Taiwan; 5 Division of Genetics and Metabolism, China Medical University Children’s Hospital, Taichung 404327, Taiwan; 6 Department of Medical Genetics, China Medical University Hospital, Taichung 404327, Taiwan; 7 Department of Medical Laboratory Science and Biotechnology, Asia University, Taichung 413305, Taiwan

**Keywords:** ClinVar, variant interpretation, allele frequency, electronic medical records, gene polymorphism, Taiwanese Han population, China Medical University Hospital Genetic Biobank

## Abstract

Background: Variant interpretation in clinical genomics requires integration of population-specific allele frequencies, curated database annotations, and phenotype evidence. However, variants annotated as pathogenic or likely pathogenic in reference databases may have different allele frequencies across populations, and electronic medical record (EMR) data may provide useful but incomplete clinical context. Methods: In this study, we used the China Medical University Hospital Genetic Biobank (CMUH-GB) and linked EMRs to evaluate ClinVar-annotated candidate variants in a Taiwanese Han population. Genotyped array variants were filtered by quality control, mapped to ClinVar, and prioritized if annotated as pathogenic or likely pathogenic and observed with an alternative allele frequency greater than 0.0001 in CMUH-GB. Results: After an updated annotation review, EMR linkage, exclusion of known rare-disease cases or ineligible loci, and retention of variants with clinically relevant EMR phenotypes, 11 candidate variants were analyzed. These variants were located in *SCN5A*, *KCNH2*, *FBP1*, *PAH*, *ACADS*, *TBX6*, *BRCA1*, *LDLR*, *GP6*, and *SLC4A11*. Several candidate variants showed substantially higher allele frequencies in CMUH-GB and the Taiwan Biobank than reported in some external population datasets. Genotype–phenotype association analyses were performed using additive genetic models with covariate adjustment and Benjamini–Hochberg false discovery rate correction. No interpretable association remained statistically significant after correction. *SCN5A* rs794728912 models for long QT syndrome and cardiac conduction defects were not estimable because no cases were observed among alternative-allele carriers, resulting in sparse-event separation. A nominal *GP6* association with coagulation defects did not remain significant after correction. These findings support population-specific reassessment prioritization of selected ClinVar-annotated variants but do not constitute formal ACMG/AMP reclassification. Conclusions: Our study highlights the value and limitations of integrating hospital-based genotyping data with EMR-derived phenotypes for ancestry-aware variant interpretation in underrepresented populations.

## 1. Introduction

Since its emergence in 2008, next-generation sequencing has transformed genomic research and clinical diagnostics. Exome sequencing and genome sequencing are now widely used to identify genetic variants potentially associated with human diseases. However, each sequencing test identifies a large number of variants, and determining which, if any, are clinically relevant remains a major challenge. A single genome may contain thousands of non-synonymous variants, many of which alter amino acid sequences but do not necessarily cause disease. Therefore, variant interpretation requires integration of population frequency, computational prediction, functional evidence, segregation data, disease mechanism, and clinical phenotype information [1,2,3].

Several bioinformatics tools and curated databases have been developed to support variant interpretation. NCBI_ClinVar [4], the Human Gene Mutation Database, and population-scale resources such as the 1000 Genomes Project provide information on reported clinical significance and allele frequencies. Computational predictors, including Sorting Intolerant from Tolerant, PolyPhen-2, and Combined Annotation Dependent Depletion, estimate the potential functional effects of variants using evolutionary conservation, protein features, and functional annotations [1,5,6]. More recently, machine learning and artificial intelligence approaches have expanded the capacity to analyze high-dimensional genomic and clinical data. For example, ensemble models such as REVEL and FATHMM integrate multiple predictive features, whereas newer deep learning tools have improved prediction of splicing effects, protein stability, and missense variant impact [7,8,9,10,11,12]. Natural language processing and other data-mining approaches can also help extract structured phenotypic information from electronic medical records (EMRs) [13,14]. Nevertheless, computational prediction remains supportive rather than definitive, and model performance may vary across ancestries and disease contexts.

Population-specific evidence is particularly important for clinical variant interpretation. Variants that are rare in one population may be more frequent in another, and such differences may affect the plausibility of a highly penetrant monogenic disease association. Allele frequency is therefore an important component of the ACMG/AMP variant interpretation framework, especially when assessed in relation to disease prevalence, inheritance mode, penetrance, and ascertainment [15,16]. However, allele frequency alone is insufficient to establish benignity or to formally reclassify a variant. Reduced penetrance, late-onset disease, recessive inheritance, variable expressivity, and incomplete clinical ascertainment may all complicate interpretation. Accordingly, population-specific allele frequencies should be considered together with disease-specific clinical evidence and curated database annotations.

This issue is especially relevant for populations that remain underrepresented in global genomic databases. Variants annotated as pathogenic or likely pathogenic in reference databases may have limited supporting evidence, conflicting submissions, or allele frequencies that differ substantially across populations [17]. Conversely, the absence of an EMR-recorded phenotype does not exclude disease, particularly for subclinical, late-onset, incompletely coded, or clinically heterogeneous conditions. Large hospital-based biobanks linked to longitudinal EMRs provide an opportunity to examine whether database annotations are consistent with local population frequencies and available phenotype records, while also requiring cautious interpretation because EMR phenotypes are not equivalent to systematic clinical screening.

In this study, we used the China Medical University Hospital Genetic Biobank (CMUH-GB), which includes large-scale genomic data from Taiwanese Han individuals linked to longitudinal EMRs, to evaluate ClinVar-annotated candidate variants prioritized for reassessment. We first identified variants annotated as pathogenic or likely pathogenic in ClinVar and observed in the CMUH-GB above a predefined allele-frequency threshold. After an updated annotation review, 11 candidate variants were retained for reassessment-oriented analysis. For these variants, we compared CMUH-GB allele frequencies with external population databases and performed EMR-based genotype–phenotype association analyses with covariate adjustment and multiple-testing correction when feasible. Our objective was not to perform formal ACMG/AMP reclassification, but to identify variants for which population-specific frequency and available EMR evidence support further disease-specific review. This approach highlights the value and limitations of integrating biobank-scale genomic data with real-world clinical records to improve variant interpretation in underrepresented populations.

## 2. Materials and Methods

### 2.1. Data Collection, Study Cohort, and Informed Consent

The Precision Medicine Project of the CMUH, Taichung, Taiwan, was initiated in 2018 to collect biospecimens and genomic data from patients receiving care at CMUH. CMUH was established in 1980, and hospital-wide electronic medical records (EMRs) have been available since 2003. Participants in the present study were recruited between 2018 and 2024. Blood samples were collected from participants who provided written informed consent. The recruitment, sample collection procedures, and clinical information collection from the EMRs of the CMUH between 2003 and 2021 were approved by the Research Ethics Committee of CMUH (CMUH-110-REC3-005 and CMUH-111-REC1-176) and were conducted in accordance with the Declaration of Helsinki.

Because participants entered the hospital system at different times and had variable durations of clinical follow-up, the available EMR observation period differed among individuals. Therefore, the absence of an EMR-recorded diagnosis was interpreted only as the absence of a recorded phenotype in the available CMUH EMR data, not as evidence that the disease was clinically absent.

### 2.2. Genotyping and Variant Quality Control

Genotyping was performed using the Taiwan Precision Medicine version 1 array (TPMv1; Thermo Fisher Scientific, Waltham, MA, USA), developed by Academia Sinica and the Taiwan Precision Medicine Initiative teams. This array contains 714,457 genotyped markers, and genotyping was performed according to the manufacturer’s protocol [18,19]. Genotype quality control was conducted using PLINK 2.0 [20]. Participants and markers were excluded using a missingness threshold of 10% per individual (--mind 0.1) and 10% per marker (--geno 0.1), respectively. Variants with a Hardy–Weinberg equilibrium (HWE) *p*-value < 1 × 10^−6^ (--hwe 10^−6^) or minor allele frequency (MAF) < 1 × 10^−4^ (--maf 0.0001) were excluded. After applying these filters, 508,004 array variants (real probes on the array) successfully passed the quality control and were retained for subsequent annotation and frequency assessment [18,19].

### 2.3. Study Population and Genetic Background

The analysis cohort included 479,724 individuals from CMUH-GB. Among these individuals, 46% were male and 54% were female, and age at study enrollment ranged from 1 to 101 years. As described in a previous overview of the CMUH-GB cohort [19], participants were predominantly Taiwanese Han individuals of East Asian ancestry. In a previous analysis of the initial 413,000 enrollees, principal component analysis showed that the cohort clustered closely with East Asian reference populations, supporting a relatively homogeneous genetic background. However, ancestry principal components were not available in the individual-level analytic datasets used for the present genotype–phenotype association analyses. Therefore, population structure was not included as a covariate in the adjusted models and was considered a residual limitation.

### 2.4. Variant Annotation and Candidate Variant Selection

The public population-frequency resources obtained from ClinVar [4], NCBI dbSNP [21], Taiwan Biobank (TWB2) [22], and GnomAD [23] were used to annotate variants and compare allele frequencies. For each genotyped marker, the reference allele frequency, alternative allele frequency (ALT AF), and minor allele frequency (MAF) were calculated in CMUH-GB. MAF was defined as the lower frequency of the two alleles in the study population, whereas ALT AF represented the frequency of the alternative allele specified in the array annotation. These measures were distinguished because the alternative allele was not necessarily the minor allele in the Taiwanese Han population.

Candidate variants were selected through a stepwise reassessment-oriented workflow. Briefly, 714,457 initial genotyped markers were filtered to 508,004 QC-passed array variants. Among these, 23,394 variants were mapped to ClinVar records, and 12,694 were annotated as pathogenic or likely pathogenic in ClinVar. Variants with CMUH-GB ALT AF >0.0001 were then prioritized for further review. After an updated annotation review, EMR linkage, exclusion of known rare-disease cases or ineligible loci, and retention of loci with clinically relevant EMR diagnoses or laboratory data available for phenotype assessment, 11 candidate variants were included in the final reassessment-oriented analysis. The complete selection workflow is provided in Supplementary Table S1.

For each candidate variant, individuals were categorized according to genotype as homozygous reference (0/0), heterozygous (0/1), or homozygous alternative (1/1). These genotypes were also converted to alternative allele dosage values of 0, 1, and 2, respectively, for regression analyses. The associated gene, reported disease condition, ClinVar clinical significance, ClinVar review status, and relevant clinical phenotypes were reviewed using ClinVar and Online Mendelian Inheritance in Man (OMIM) [24]. External allele-frequency comparisons were performed using Taiwan Biobank and public population databases when available. This process was intended to prioritize variants for further disease-specific reassessment and did not constitute formal ACMG/AMP reclassification.

### 2.5. EMR Phenotype Extraction and Manual Review

For each candidate gene, disease-related EMR phenotypes were defined using available structured diagnosis records, laboratory measurements, anthropometric data, or cancer diagnosis records, depending on the expected clinical phenotype. Examples included long QT syndrome and cardiac conduction defects for *SCN5A* and *KCNH2*; fasting glucose and hypoglycemia for *FBP1*; acidosis-related diagnosis codes for *PAH* and *ACADS*; adult height and short-stature definitions for *TBX6*; breast cancer diagnosis in female individuals for *BRCA1*; LDL cholesterol and elevated LDL cholesterol for *LDLR*; coagulation-related diagnoses for *GP6*; and corneal dystrophy for *SLC4A11*. Phenotype definitions, EMR data sources, and phenotype ascertainment limitations are summarized in Supplementary Table S4.

For variants in which homozygous alternative carriers were rare, available EMRs were manually reviewed to determine whether recorded diagnoses, symptoms, laboratory findings, or clinical histories were consistent with the reported disease phenotypes. These manual reviews were descriptive and were not used as evidence for formal reclassification. Because EMRs were collected during routine clinical care rather than through systematic disease screening, incomplete ascertainment, subclinical disease, late-onset manifestations, and variable follow-up duration were considered when interpreting negative EMR findings.

### 2.6. Statistical Analysis

Primary genotype–phenotype association analyses were performed using individual-level data for the 11 candidate variants. For each variant, genotypes were coded using an additive genetic model according to the number of alternative alleles: 0/0 = 0, 0/1 = 1, and 1/1 = 2. Effect estimates therefore represent the association per additional alternative allele.

Binary EMR phenotypes were coded as 0 for controls and 1 for cases. Records coded with no data or missing were excluded from the corresponding phenotype-specific analysis. Quantitative traits included fasting glucose, adult height, and LDL cholesterol. Implausible or invalid values were treated as missing. Adult height analyses for *TBX6* were restricted to individuals older than 20 years and were performed separately in males and females, with adjustment for age only. The *TBX6* short-stature phenotype was analyzed in the combined cohort because it had been defined using sex-specific criteria, and the model was adjusted for age and sex. *BRCA1* breast cancer analysis was restricted to female individuals aged ≥ 20 years and was adjusted for age only.

For binary outcomes, logistic regression was used to estimate the odds ratio (OR) and 95% confidence interval (CI) per additional alternative allele, adjusting for age and sex when applicable. For quantitative outcomes, linear regression was used to estimate the beta coefficient and 95% CI per additional alternative allele. For sparse binary outcomes or models with separation, Firth logistic regression or bias-reduced logistic regression was attempted when available. If a model did not converge, produced complete or quasi-complete separation, generated non-finite estimates, or yielded non-interpretable estimates such as boundary ORs or invalid confidence intervals, the adjusted effect estimate was reported as not estimable and was excluded from multiple-testing correction.

Multiple-testing correction was performed across the interpretable primary genotype–phenotype association tests using the Benjamini–Hochberg false discovery rate (FDR) procedure. Both raw *p* values and FDR q values were reported. Models with non-interpretable estimates were assigned missing *p* values and were not included in the FDR calculation. Statistical analyses were performed using R. The results were interpreted as covariate-adjusted EMR-based association analyses to support reassessment prioritization, not as definitive evidence for or against variant pathogenicity.

## 3. Results

### 3.1. Selection and Annotation of Candidate Variants

A stepwise reassessment-oriented workflow was used to identify candidate variants for population-specific evaluation. Among 714,457 initial genotyped markers on the TPMv1 array, 508,004 variants passed genotype quality control. Of these, 23,394 variants were mapped to ClinVar records, and 12,694 were annotated as pathogenic or likely pathogenic. Variants with an alternative allele frequency (ALT AF) greater than 0.0001 in the CMUH-GB were prioritized for further review. After EMR linkage, exclusion of known rare-disease cases or ineligible loci, and retention of variants with clinically relevant EMR diagnoses or laboratory data available for phenotype assessment, 11 candidate variants were retained for final analysis (Supplementary Table S1).

The 11 candidate variants were located in *SCN5A*, *KCNH2*, *FBP1*, *PAH*, *ACADS*, *TBX6*, *BRCA1*, *LDLR*, *GP6*, and *SLC4A11* (Table 1). These variants were associated with cardiac arrhythmia or conduction phenotypes, long QT syndrome, fructose-1,6-bisphosphatase deficiency, PAH-related metabolic disease, acyl-CoA dehydrogenase-related disorders, spondylocostal dysostosis, hereditary breast and ovarian cancer susceptibility, familial hypercholesterolemia, platelet-type bleeding disorder, and corneal endothelial dystrophy (Table 1 and Supplementary Table S3). Because several retained loci were deletion, insertion, multi-nucleotide, or complex delins variants, they are referred to as candidate array variants rather than exclusively as SNVs.

### 3.2. Genotype Quality and Allele-Frequency Comparisons

All 11 candidate variants were directly genotyped by array probes and had low missing rates, ranging from 0.04% to 0.44% (Supplementary Table S2). Several variants annotated as pathogenic, likely pathogenic, or conflicting in external databases were observed at relatively high frequencies in CMUH-GB. The CMUH-GB minor allele frequencies were 0.135140 for *KCNH2* rs1060500661, 0.134988 for *KCNH2* rs199473518, 0.041373 for *FBP1* rs1057517733, 0.243386 for *PAH* rs796052017, 0.448550 for *ACADS* rs796051906, 0.463121 for *TBX6* rs3809627, 0.384015 for *LDLR* rs1555805360, 0.157269 for *GP6* rs754929349, and 0.412258 for *SLC4A11* rs797045107 (Table 1 and Supplementary Table S2). In contrast, *SCN5A* rs794728912 and *BRCA1* rs886040027 were relatively uncommon, with minor allele frequencies of 0.000339 and 0.001368, respectively.

Allele-frequency comparisons with Taiwan Biobank showed broadly similar frequencies for variants with available Taiwan Biobank data, including *KCNH2*, *PAH*, *ACADS*, *TBX6*, *LDLR*, *GP6*, and *SLC4A11* variants (Table 1 and Supplementary Tables S2 and S3). For several variants, external global or East Asian frequencies were absent or substantially lower than the frequencies observed in CMUH-GB and Taiwan Biobank, supporting the need for population-specific reassessment rather than direct inference of pathogenicity (Supplementary Table S3).

### 3.3. Manual Review of Rare Homozygous Alternative Carriers

Two variants had only one homozygous alternative carrier in the CMUH-GB genotype data: *SCN5A* rs794728912 and *BRCA1* rs886040027 (Supplementary Table S2). Manual EMR review of the single *SCN5A* homozygous alternative carrier identified a 59-year-old male with recorded diagnoses including cough and malignant neoplasm of the rectum, without documented long QT syndrome or cardiac conduction disease in the available CMUH EMR. Manual EMR review of the single *BRCA1* homozygous alternative carrier identified a 69-year-old male with recorded diagnoses including chest pain, colic, and diverticulitis of the large intestine, without documented breast or gonadal cancer. These observations were descriptive only and were not used as evidence for formal variant reclassification.

### 3.4. EMR Phenotype Definitions and Adjusted Association Analyses

EMR-based phenotypes were defined according to the expected disease spectrum of each candidate gene. Cardiac phenotypes for *SCN5A* and *KCNH2* included long QT syndrome and all cardiac conduction defects. *FBP1* was evaluated using fasting glucose and hypoglycemia; *PAH* and *ACADS* using acidosis-related diagnosis codes; *TBX6* using adult height and short stature; *BRCA1* using breast cancer records in female individuals aged ≥ 20 years; *LDLR* using LDL cholesterol and elevated LDL cholesterol; *GP6* using coagulation-related diagnosis codes; and *SLC4A11* using corneal dystrophy diagnosis codes (Supplementary Table S4). These EMR phenotypes were interpreted as operational definitions because routine EMR data may incompletely capture subclinical disease, late-onset manifestations, family history, medication exposure, confirmatory biochemical testing, platelet function studies, or ophthalmologic findings.

Primary genotype–phenotype association analyses were performed using an additive genetic model, with genotypes coded according to the number of alternative alleles. Binary EMR phenotypes were analyzed using logistic regression, Firth logistic regression, or bias-reduced logistic regression when required for sparse outcomes, whereas quantitative traits were analyzed using linear regression. Models were adjusted for age and sex when applicable. *TBX6* adult height analyses were performed separately in males and females older than 20 years with age adjustment only, and *TBX6* short stature was analyzed in the combined cohort with age and sex adjustment. *BRCA1* breast cancer analysis was restricted to female individuals aged ≥ 20 years and adjusted for age only. Interpretable primary tests were corrected using the Benjamini–Hochberg false discovery rate procedure (Table 2).

For *SCN5A* rs*794728912*, adjusted models for long QT syndrome and all cardiac conduction defects were not estimable because of sparse events and genotype separation. No cases were observed among heterozygous or homozygous alternative-allele carriers in either phenotype analysis. Because all cases occurred among reference homozygotes, adjusted odds ratios and *p* values were not interpretable; these models were excluded from FDR correction (Table 2).

For the two *KCNH2* variants, no significant association was observed with long QT syndrome or all cardiac conduction defects after covariate adjustment and FDR correction. The long QT analyses were sparse and were therefore interpreted cautiously. Similarly, no significant association remained after FDR correction for *FBP1*-related fasting glucose or hypoglycemia, *PAH*- or *ACADS*-related acidosis, *TBX6*-related adult height or short stature, *BRCA1*-related breast cancer, *LDLR*-related LDL cholesterol or elevated LDL cholesterol, or *SLC4A11*-related corneal dystrophy (Table 2). For *LDLR* rs1555805360, the adjusted LDL-C effect size was small, supporting limited clinical relevance in this dataset. For *PAH* and *ACADS*, acidosis was treated as a nonspecific proxy phenotype rather than a disease-specific metabolic diagnosis (Supplementary Table S4).

*GP6* rs754929349 showed a nominal per-alternative-allele association with coagulation defects, with an OR below 1 and a raw *p* value below 0.05. However, this association did not remain significant after FDR correction and was interpreted cautiously because mild bleeding tendency may be underdiagnosed and platelet function testing was not systematically available (Table 2 and Supplementary Table S4).

Overall, none of the interpretable genotype–phenotype associations remained statistically significant after covariate adjustment and FDR correction. The two *SCN5A* analyses were not estimable because of sparse-event separation, and the nominal *GP6* finding did not remain significant after multiple-testing correction. These results were used to support population-specific reassessment prioritization and were not considered formal ACMG/AMP variant reclassification.

## 4. Discussion

This study used large-scale genotyping data from the CMUH-GB, together with longitudinal EMR information, to evaluate ClinVar-annotated candidate variants in the Taiwanese Han population. After updated annotation review, 11 candidate variants were retained for reassessment-oriented analysis. Several of these variants showed substantially higher allele frequencies in CMUH-GB than expected for highly penetrant monogenic disease-causing variants, and their allele frequencies were broadly consistent with those observed in Taiwan Biobank. These findings emphasize the importance of incorporating population-specific allele-frequency data into variant interpretation, particularly for populations that remain underrepresented in global genomic databases.

Allele frequency is an important component of variant interpretation, but it is not sufficient by itself to establish benignity or to formally reclassify a variant. The ACMG/AMP framework considers multiple categories of evidence, including population frequency, predicted or experimentally demonstrated functional impact, segregation, de novo occurrence, allelic data, inheritance mode, disease prevalence, penetrance, phenotype specificity, and case-level evidence [25]. Our study provides two major types of evidence: allele-frequency data from a large Taiwanese Han biobank and EMR-based phenotype observations. These data are useful for identifying variants that warrant further review, but they do not provide a complete ACMG/AMP evidence set. Therefore, the present results should be interpreted as supporting population-specific reassessment prioritization rather than formal reclassification.

The ancestry-specific distribution of genetic variation is a central issue in clinical genomics. Variants that are rare in one population may be more frequent in another, and the clinical interpretation of such variants depends on disease prevalence, inheritance pattern, penetrance, and ascertainment [17]. In this study, several candidate variants had relatively high frequencies in CMUH-GB and similar frequencies in Taiwan Biobank, suggesting that they may represent population-enriched alleles in Taiwanese Han individuals. Such observations do not by themselves exclude disease relevance, especially for recessive disorders, reduced penetrance, mild phenotypes, or late-onset conditions. However, they do raise questions about whether these variants are consistent with the expected effect size and disease mechanism implied by a pathogenic or likely pathogenic annotation.

The candidate variants evaluated in this study also differ in gene function, variant type, inheritance pattern, and expected disease mechanism. Variants in cardiac ion-channel genes such as *SCN5A* and *KCNH2* require interpretation in relation to electrophysiologic phenotypes, penetrance, medication exposure, and arrhythmia history. Variants in metabolic genes such as *PAH*, *FBP1*, and *ACADS* may require biochemical testing and disease-specific metabolic data rather than nonspecific EMR proxy diagnoses. Cancer-predisposition and lipid-related genes such as *BRCA1* and *LDLR* require consideration of age-dependent penetrance, sex-specific risk, family history, tumor spectrum, and quantitative lipid effects. Therefore, the 11 candidate variants should not be interpreted using a single uniform mechanism. Our analysis provides population-frequency and EMR-based observational evidence, but it does not replace transcript-specific annotation, protein-impact prediction, splicing assessment, functional assays, segregation analysis, or disease-specific expert curation [26].

The EMR-based genotype–phenotype analyses further supported cautious interpretation. In the revised association analyses, no interpretable genotype–phenotype association remained statistically significant after covariate adjustment and Benjamini–Hochberg false discovery rate correction. The two *SCN5A* rs794728912 analyses for long QT syndrome and all cardiac conduction defects were not estimable because no cases were observed among heterozygous or homozygous alternative-allele carriers, resulting in sparse-event separation and model non-convergence. This finding should not be interpreted as evidence of a protective effect or absence of cardiac disease, but rather as a limitation of statistical estimation in a very rare carrier group. The nominal association observed for *GP6* rs754929349 with coagulation defects did not remain significant after FDR correction and requires disease-specific validation. For *LDLR* rs1555805360, the adjusted effect size for LDL cholesterol was small, and the association did not remain significant after correction. For *PAH*, *ACADS*, *BRCA1*, *SLC4A11*, *TBX6*, *FBP1*, and *KCNH2*, the available EMR-based analyses did not identify robust associations after correction.

The *PAH* rs796052017 finding illustrates the need to combine local population frequency with disease-specific clinical context. *PAH* is associated with phenylalanine metabolism disorders, including phenylketonuria. In this study, rs796052017 was observed at a high frequency in CMUH-GB and the Taiwan Biobank, but the EMR-based acidosis phenotype used for analysis was only a nonspecific proxy and was not equivalent to biochemical diagnosis of PAH deficiency. Importantly, Taiwan has implemented newborn screening since 1985, including screening for phenylketonuria. In addition, the molecular study of Taiwanese PAH-deficient patients reported by Liang et al. [27] did not identify rs796052017 among reported *PAH* variants. Together, these observations suggest that rs796052017 does not appear to be a recognized common cause of clinically detected PAH deficiency in Taiwan. Nevertheless, because our study did not include systematic phenylalanine measurements, newborn screening records, dietary treatment information, or confirmatory metabolic testing, this observation should be considered supportive of further population-specific reassessment rather than definitive reclassification.

The integration of genotyping and EMR data is a strength of this study. Large-scale hospital-based biobanks provide an opportunity to evaluate whether variant annotations derived from global or heterogeneous databases are consistent with local allele frequencies and available clinical records. By linking CMUH-GB genotypes with EMRs, we were able to examine both population frequency and clinically relevant phenotype proxies for 11 candidate variants. This approach is particularly valuable for underrepresented populations, in which reference databases may not adequately capture local allele-frequency distributions. Our findings indicate that several ClinVar-annotated candidate variants observed in Taiwanese Han individuals merit further expert review, especially when their local frequencies are difficult to reconcile with a highly penetrant monogenic disease model. Moving forward, the integration of large-scale genomic biobanks with real-world medical records may provide important population-specific evidence to support variant interpretation and reassessment. Our findings reinforce the need for localized genomic studies and disease-specific clinical validation to improve the contextual interpretation of genetic variants in diverse and underrepresented populations [28,29].

This study has several limitations. First, the EMR data were collected during routine clinical care and were not generated through systematic screening for each disease phenotype. Therefore, the absence of an EMR-recorded diagnosis cannot be interpreted as an absence of disease. Subclinical phenotypes, mild manifestations, late-onset disease, incomplete coding, outside-hospital diagnoses, and variable follow-up duration may have led to underascertainment. Second, recruitment into the CMUH Precision Medicine Project began in 2018, whereas the extracted EMR period covered 2003–2021; individual participants differed in the duration and intensity of clinical observation. Third, several phenotypes were based on operational EMR definitions or proxy diagnoses. For example, acidosis was not disease-specific for PAH or ACADS deficiency, and cardiac, ophthalmologic, platelet-function, cancer-family-history, biochemical, and treatment data were not systematically available for all participants. Fourth, ancestry principal components were not available in the individual-level association dataset, so residual population stratification cannot be fully excluded, although the cohort is predominantly Taiwanese Han and has previously been shown to cluster with East Asian reference populations. Fifth, our analyses focused on array-genotyped variants and did not include comprehensive sequencing, segregation analysis, functional assays, or curated case-level evidence. These limitations mean that our findings should not be used as stand-alone evidence for ACMG/AMP reclassification.

## 5. Conclusions

In conclusion, this study demonstrates the value of integrating population-specific genotyping data with EMR-based phenotype information to prioritize ClinVar-annotated variants for further review in the Taiwanese Han population. The revised analyses did not identify robust genotype–phenotype associations after covariate adjustment and multiple-testing correction, and several candidate variants showed population frequencies that warrant careful reassessment in an ancestry-specific context. These findings support the need for expanded Taiwanese and East Asian reference data, disease-specific clinical validation, and ACMG/AMP-guided expert curation before any formal change in variant classification is considered.

## Figures and Tables

**Table 1:** Candidate ClinVar-Annotated Variants Prioritized for Reassessment in the Taiwanese Han Population.

Gene	rsID	Chr:Position_GRCh38	REFALT	Variant Type	HGVS	CMUH-GB ALT AF	CMUH-GB MAF	TWB2 ALT AF	gnomAD EAS AF	gnomAD Global AF	ClinVar Clinical Significance	ClinVar Review Status	ClinVar Associated Condition	Rationale for Reassessment Prioritization	Interpretation in This Study
SCN5A	rs794728912	chr3:38585944	AC>A	deletion	NM_000335.5:c.2533del (p.Val845fs)	0.0003	0.0003	0.0005	NA	NA	Pathogenic/Likely pathogenic	criteria provided, multiple submitters, no conflicts	Cardiovascular phenotype; Cardiac arrhythmia; not provided; Brugada syndrome; Brugada syndrome 1	Discordance between ClinVar annotation and population frequency	Candidate for reassessment; not formally reclassified
KCNH2	rs1060500661	chr7:150951697	AG>A	deletion	NM_000238.4:c.1695del (p.Cys566fs)	0.1351	0.1351	0.1339	0	0.0000007	Pathogenic	criteria provided, single submitter	Long QT syndrome	Discordance between ClinVar annotation and population frequency	Candidate for reassessment; not formally reclassified
KCNH2	rs199473518	chr7:150951700	C>G	SNV	NM_000238.4:c.1693G>C (p.Ala565Pro)	0.8650	0.1350	0.8665	NA	NA	Likely pathogenic	criteria provided, multiple submitters, no conflicts	Cardiovascular phenotype; not provided	Population-specific allele frequency suggests need for reassessment	Candidate for reassessment; not formally reclassified
FBP1	rs1057517733	chr9:94603438	T>CC	delins/complex	NM_000507.4:c.960delinsGG (p.Ser321fs)	0.9586	0.0414	0.9595	NA	NA	Pathogenic	criteria provided, multiple submitters, no conflicts	not provided; Fructose-biphosphatase deficiency	Population-specific allele frequency suggests need for reassessment	Candidate for reassessment; not formally reclassified
PAH	rs796052017	chr12:102852922	CA>TG	MNV	NM_000277.1:c.734_735inv (p.Val245Ala)	0.7566	0.2434	0.759	NA	NA	NA	NA	NA	External annotation unavailable; requires manual verification; High population-specific allele frequency	Candidate for reassessment; not formally reclassified
ACADS	rs796051906	chr12:120738874	CGC>TGT	delins/complex	NM_000017.4:c.988_990delinsTGT (p.Arg330Cys)	0.5515	0.4486	0.5502	NA	NA	Pathogenic/Likely pathogenic	criteria provided, multiple submitters, no conflicts	ACADS-related disorder; not provided; Deficiency of butyryl-CoA dehydrogenase	Population-specific allele frequency suggests need for reassessment	Candidate for reassessment; not formally reclassified
TBX6	rs3809627	chr16:30091839	C>A	SNV	NM_004608.4:c.-49+34G>T	0.5369	0.4631	0.5419	0.5713	0.4146	Conflicting classifications of pathogenicity; Pathogenic	criteria provided, conflicting classifications; no assertion criteria provided	not provided; Fetal anomalies with a likely genetic cause; not specified; Spondylocostal dysostosis 5	Discordance between ClinVar annotation and population frequency	Candidate for reassessment; not formally reclassified
BRCA1	rs886040027	chr17:43093184	->A	insertion	NM_007294.4:c.2346dup (p.Ile783fs)	0.0014	0.0014	0	NA	NA	Pathogenic	reviewed by expert panel	Breast-ovarian cancer, familial, susceptibility to, 1	Discordance between ClinVar annotation and population frequency	Candidate for reassessment; not formally reclassified
LDLR	rs1555805360	chr19:11113589	AG>GGACAT	delins/complex	NM_000527.5:c.1413_1414delinsGGACAT (p.Gln474fs)	0.6160	0.3840	0.62	NA	NA	Pathogenic/Likely pathogenic	criteria provided, multiple submitters, no conflicts	Cardiovascular phenotype; Familial hypercholesterolemia; not provided	Population-specific allele frequency suggests need for reassessment	Candidate for reassessment; not formally reclassified
GP6	rs754929349	chr19:55018664	CTTCG>C	deletion	NM_016363.5:c.708_711del (p.Asn236fs)	0.1573	0.1573	0.155	0.0000892	0.0001207	Pathogenic/Likely pathogenic	criteria provided, multiple submitters, no conflicts	Platelet-type bleeding disorder 11	Discordance between ClinVar annotation and population frequency	Candidate for reassessment; not formally reclassified
SLC4A11	rs797045107	chr20:3234173	TGGCGAAGC>G	delins/complex	NM_001174089.2:c.425_433delinsC (p.Arg142fs)	0.5877	0.4123	0.5877	NA	NA	Pathogenic	criteria provided, multiple submitters, no conflicts	Corneal dystrophy, Fuchs endothelial, 4; Corneal dystrophy-perceptive deafness syndrome; Congenital hereditary endothelial dystrophy of cornea; not provided	Population-specific allele frequency suggests need for reassessment	Candidate for reassessment; not formally reclassified

Chr, chromosome number; REF, reference allele; ALT, alternative allele; AF, allele frequency. TWB2 website information: https://taiwanview.twbiobank.org.tw (Accessed on 30 May 2026). gnomAD website information: https://gnomad.broadinstitute.org/ (Accessed on 30 May 2026). ClinVar website information: https://www.ncbi.nlm.nih.gov/clinvar/ (Access on 30 May 2026). dbSNP website information: https://www.ncbi.nlm.nih.gov/snp/ (Accessed on 30 May 2026).

**Table 2:** Summary of Covariate-Adjusted Genotype–Phenotype Association Analyses.

Gene	rsID	Phenotype	Outcome Type	Analysis Population	Genetic Model	Statistical Model	Covariates	N Analyzed	Cases/Controls or Trait Summary	Effect Estimate	95% CI	Raw *p* Value	FDR q Value	Interpretation
SCN5A	rs794728912	Long QT syndrome	Binary EMR phenotype	All eligible individuals	Additive model, per ALT allele	Bias-reduced logistic regression; not converged or not interpretable	Age, sex	434608	cases = 43; controls = 434,565	Not estimable	Not estimable	NA	NA	Adjusted estimate was not interpretable because of sparse events and model non-convergence; no definitive association inference was made. Absence of coded diagnosis does not exclude subclinical cardiac phenotypes.
SCN5A	rs794728912	All cardiac conduction defects	Binary EMR phenotype	All eligible individuals	Additive model, per ALT allele	Bias-reduced logistic regression; not converged or not interpretable	Age, sex	434608	cases = 325; controls = 434,283	Not estimable	Not estimable	NA	NA	Adjusted estimate was not interpretable because of sparse events and model non-convergence; no definitive association inference was made. Absence of coded diagnosis does not exclude subclinical cardiac phenotypes.
KCNH2	rs1060500661	Long QT syndrome	Binary EMR phenotype	All eligible individuals	Additive model, per ALT allele	Bias-reduced logistic regression	Age, sex	432870	cases = 42; controls = 432,828	OR per ALT allele = 1.10	(0.61, 1.99)	0.7435	0.7930	No significant association after FDR correction; sparse events limit interpretation. Absence of coded diagnosis does not exclude subclinical cardiac phenotypes.
KCNH2	rs1060500661	All cardiac conduction defects	Binary EMR phenotype	All eligible individuals	Additive model, per ALT allele	Logistic regression	Age, sex	432870	cases = 322; controls = 432,548	OR per ALT allele = 0.89	(0.70, 1.12)	0.3207	0.6415	No significant association after covariate adjustment and FDR correction. Absence of coded diagnosis does not exclude subclinical cardiac phenotypes.
KCNH2	rs199473518	Long QT syndrome	Binary EMR phenotype	All eligible individuals	Additive model, per ALT allele	Bias-reduced logistic regression	Age, sex	432980	cases = 43; controls = 432,937	OR per ALT allele = 1.18	(0.67, 2.08)	0.5751	0.7606	No significant association after FDR correction; sparse events limit interpretation. Absence of coded diagnosis does not exclude subclinical cardiac phenotypes.
KCNH2	rs199473518	All cardiac conduction defects	Binary EMR phenotype	All eligible individuals	Additive model, per ALT allele	Logistic regression	Age, sex	432980	cases = 325; controls = 432,655	OR per ALT allele = 0.91	(0.72, 1.15)	0.4092	0.7274	No significant association after covariate adjustment and FDR correction. Absence of coded diagnosis does not exclude subclinical cardiac phenotypes.
FBP1	rs1057517733	Fasting glucose	Quantitative laboratory trait	All eligible individuals	Additive model, per ALT allele	Linear regression	Age, sex	227136	mean = 90.04, SD = 21.90	Beta per ALT allele = 0.07	(−0.25, 0.39)	0.6655	0.7606	No significant association after covariate adjustment and FDR correction.
FBP1	rs1057517733	Hypoglycemia	Binary EMR phenotype	All eligible individuals	Additive model, per ALT allele	Logistic regression	Age, sex	227140	cases = 20,350; controls = 206,790	OR per ALT allele = 1.00	(0.96, 1.06)	0.8511	0.8511	No significant association after covariate adjustment and FDR correction.
PAH	rs796052017	Acidosis	Binary EMR phenotype	All eligible individuals	Additive model, per ALT allele	Logistic regression	Age, sex	434335	cases = 2571; controls = 431,764	OR per ALT allele = 0.96	(0.90, 1.02)	0.2049	0.5870	No significant association after covariate adjustment and FDR correction. Acidosis is a nonspecific proxy phenotype and requires disease-specific validation.
ACADS	rs796051906	Acidosis	Binary EMR phenotype	All eligible individuals	Additive model, per ALT allele	Logistic regression	Age, sex	434319	cases = 2822; controls = 431,497	OR per ALT allele = 0.99	(0.94, 1.04)	0.5945	0.7606	No significant association after covariate adjustment and FDR correction. Acidosis is a nonspecific proxy phenotype and requires disease-specific validation.
TBX6	rs3809627	Adult height	Quantitative anthropometric trait	Male only; Age >20	Additive model, per ALT allele	Linear regression	Age only	89247	mean = 169.40, SD = 6.26	Beta per ALT allele = 0.03	(−0.03, 0.08)	0.3039	0.6415	No significant association with adult height after age adjustment and FDR correction.
TBX6	rs3809627	Adult height	Quantitative anthropometric trait	Female only; Age >20	Additive model, per ALT allele	Linear regression	Age only	103150	mean = 158.41, SD = 6.35	Beta per ALT allele = 0.01	(−0.04, 0.06)	0.6225	0.7606	No significant association with adult height after age adjustment and FDR correction.
TBX6	rs3809627	Short stature	Binary EMR phenotype	All eligible individuals	Additive model, per ALT allele	Logistic regression	Age, sex	192889	cases = 9183; controls = 183,706	OR per ALT allele = 1.02	(0.99, 1.05)	0.2010	0.5870	No significant association with sex-specific short-stature phenotype after covariate adjustment and FDR correction.
BRCA1	rs886040027	Breast cancer	Binary EMR phenotype	Female only; Age > = 20	Additive model, per ALT allele	Logistic regression	Age only; female-only analysis	208081	cases = 9954; controls = 198,127	OR per ALT allele = 0.87	(0.58, 1.30)	0.5050	0.7606	No significant association after covariate adjustment and FDR correction. EMR-based breast cancer analysis is insufficient for pathogenicity interpretation.
LDLR	rs1555805360	LDL cholesterol	Quantitative laboratory trait	All eligible individuals	Additive model, per ALT allele	Linear regression	Age, sex	139337	mean = 129.31, SD = 37.92	Beta per ALT allele = 0.27	(−0.01, 0.56)	0.0628	0.5028	No significant association after covariate adjustment and FDR correction; the adjusted LDL-C effect size was small.
LDLR	rs1555805360	Elevated LDL cholesterol	Binary EMR phenotype	All eligible individuals	Additive model, per ALT allele	Logistic regression	Age, sex	139337	cases = 64,472; controls = 74,865	OR per ALT allele = 1.01	(0.99, 1.03)	0.2201	0.5870	No significant association after covariate adjustment and FDR correction.
GP6	rs754929349	Coagulation defects	Binary EMR phenotype	All eligible individuals	Additive model, per ALT allele	Logistic regression	Age, sex	434085	cases = 1771; controls = 432,314	OR per ALT allele = 0.91	(0.82, 0.99)	0.0367	0.5028	Nominal per-ALT-allele association with a small effect size; not significant after FDR correction and requires disease-specific validation.
SLC4A11	rs797045107	Corneal dystrophy	Binary EMR phenotype	All eligible individuals	Additive model, per ALT allele	Logistic regression	Age, sex	433889	cases = 819; controls = 433,070	OR per ALT allele = 0.94	(0.85, 1.04)	0.2180	0.5870	No significant association after covariate adjustment and FDR correction. Ophthalmologic underascertainment remains possible.

OR, odds ratio; 95% confidence interval, 95% CI; FDR, Benjamini–Hochberg false discovery rate. Genotypes were coded as 0, 1, and 2 according to the number of alternative alleles, corresponding to REF/REF, REF/ALT, and ALT/ALT, respectively. Effect estimates represent the change in outcome per additional ALT allele.

## Data Availability

Data are provided within the manuscript and Supplementary Information files. Additional data may be made available from the corresponding author upon reasonable request and with appropriate approval.

## Supplementary Materials

The following supporting information can be downloaded at: https://www.mdpi.com/article/10.3390/genes17070818/s1, Supplementary Tables S1–S4.
